# On Functional Derangements of the Liver

**Published:** 1875-08

**Authors:** 


*On Functional Derangements of the Liver.* Being the Croonian Lectures delivered at the Royal College of Physicians, in March, 1874. By Charles E. Murchison, M. D., LL. D., F. R. S. New York: Wm. Wood & Co., 1875. Buffalo: H. H. Otis. pp. XVI, 182.

The author says that these lectures do not pretend to place the subject upon a firm and lasting basis; but by calling attention to its importance and provoking discussion, it is hoped that they will prove a stepping-stone to a more certain knowledge of it, and in the mean time that they will help to supply what appears to be a deficiency in medical literature. The opening lecture gives a sketch of the present knowledge of the physiology of the liver, which is an excellent resumé of the subject which shows a wide range of reading and a thorough acquaintance with the subject. Following this is a classification of the functional derangements of that organ. This classification is based upon the normal functions of the gland and upon the symptoms which a disordered liver may produce in the different physiological systems of the body. The derangements are classed under nine separate heads, viz :—abnormal nutrition, elimination and disintegration; derangements of the organs of digestion, of the nervous system, of the organs of circulation, of respiration and of the urinary organs, and abnormal conditions of the skin.

The discussion of these different subjects are of more than usual interest, and show the work of a thorough student.

In view of what has been said in the notice of the work by Sir Henry Thompson in this issue upon the agency of functional derangements of the liver in the production of urinary deposits, the remarks of Dr. Murchison upon abnormal disintegration are of especial interest. The author prefers in designating an excess of uric acid and of the urates to employ the term lithæmia in preference to uricæmia as proposed by Dr. Austin Flint.

Dr. Murchison is particularly emphatic in saying that this condition of lithæmia is not due to a derangement of the function of the kidneys but rather of the liver, of a more or less temporary character. Among the effects of the abnormal disintegration, are mentioned urinary and biliary calculi, gout, degeneration of the tissues and the derangements of the liver, which are named next in order in his classification.

The volume is closed with a consideration of the causes and treatment of derangements of the liver. Dr. Murchison has succeeded admirably in placing this subject before the profession in an instructive and suggestive form. It will doubtless be the means of stimulating others to investigation and study in this comparatively unknown region, and we hope that the author, encouraged by the success of this effort, will give to the profession the result of this studies in a still more comprehensive form. The book is printed on tinted paper in clear type and handsomely bound. No student or practitioner of medicine should be without a clear understanding of its contents.

